# Time Trends of Percutaneous Injuries in Hospital Nurses: Evidence of the Interference between Effects of Adoption of Safety Devices and Organizational Factors

**DOI:** 10.3390/ijerph18084371

**Published:** 2021-04-20

**Authors:** Marco M. Ferrario, Giovanni Veronesi, Rossana Borchini, Marco Cavicchiolo, Oriana Dashi, Daniela Dalla Gasperina, Giovanna Martinelli, Francesco Gianfagna

**Affiliations:** 1 Department of Medicine and Surgery, School of Medicine, University of Insubria, 21100 Varese, Italy; giovanni.veronesi@uninsubria.it (G.V.); d.dallagasperina@uninsubria.it (D.D.G.); francesco.gianfagna@uninsubria.it (F.G.); 2 Occupational, Preventive and Toxicology Unit, ASST Sette Laghi, 21100 Varese, Italy; 3 Occupational and Preventive Medicine Unit, ASST Lariana, 22100 Como, Italy; rossana.borchini@asst-lariana.it; 4 School of Specialization in Occupational Medicine, University of Insubria, 21100 Varese, Italy; m.cavicchiolo@studenti.uninsubria.it (M.C.); oriana.dashi@gmail.com (O.D.); 5 Quality Health Assessment Unit, ASST Sette Laghi, 21100 Varese, Italy; giovanna.martinelli@asst-settelaghi.it; 6 Mediterranea Cardiocentro, 80122 Napoli, Italy

**Keywords:** needlestick injuries, nurses, protective devices, shift work schedule, personnel administration, hospital

## Abstract

Few studies have focused on the combined effects of devices and work organization on needlestick injuries trends. The aim of the study was to estimate trends of percutaneous injury rates (IR) in nurses (N) and nurse assistants (NA) over a 10 year period, in which passive safety devices were progressively adopted. Percutaneous and mucocutaneous injuries registered in a University Hospital in Northern Italy in Ns and NAs in 2007–2016 were analyzed. Organizational data were also available on shift schedules, turnover, downsizing and age- and skill-mix. We estimated IRs per 100 full-time equivalent workers from Poisson models and their average annual percent changes (APC) from joinpoint regression model. In the entire period, monotonic decreases in percutaneous IRs occurred among day-shift Ns (APC = −20.9%; 95% CI: −29.8%, −12%) and NAs (APC = −15.4%; −32.9%, 2.2%). Joinpoint modeling revealed a turning point in 2012 for night-shift Ns, with a steady decline in 2007–2012 (APC = −19.4%; −27.9%, −10.9%), and an increase thereafter (APC = +13.5%; 1.5%, 25.5%). In comparison to 2008 and 2012, in 2016 night-shift Ns were 5.9 and 2.5 times more likely to be younger and less qualified or experienced than day-shift Ns. The observed declines in percutaneous injury rates occurred in a time period when safety devices were progressively implemented. The causal nature of multiple exposures and organizational procedures in affecting injury time trends should be further addressed by quasi-experimental studies.

## 1. Introduction

Occupational exposures to blood-borne infections are one of the most frequent accidents occurring in healthcare settings. Percutaneous injuries (PI), occurring mainly while using or disposing of needles and in smaller proportions of scalpels or other sharp devices, occur far more frequently than accidental contacts with blood or other body fluids through bare skin or mucous membranes (mucocutaneous exposures) [1,2]. Needlestick injury (NSI) rates in nurses are reported to vary between 2.1 and 5.5 per 100 full-time equivalent (FTE) in different studies [3,4,5,6,7,8]. The introduction of safety devices has been shown to lower NSI rates [9,10,11,12], although recent meta-analyses support evidence of the effectiveness in prevention of NSI events of passive intravenous systems only, i.e., safety containers which allow avoiding needle recap; but not for devices with more sophisticated active safe mechanisms [13,14]. Besides, to reduce the risk associated with the NSI, international guidelines emphasize the need to develop specific training for all health workers [15].

Shift work and sleep deprivation in healthcare workers have been linked to decreased performance and increased occupational injuries as well as clinical errors [16,17,18,19,20,21]. In particular, literature reported higher rates of any kind of occupational injuries [22,23] and an increased risk of NSIs [24,25] among nurses working on night shifts or being on irregular shift schedules. Among hospital nurses, different shift-level staffing models have been found to influence patient- and nurse-related outcomes, including occupational accidents and NSIs [26,27,28,29]. A lower-level experience of the nurse staff seems to be associated with higher NSI rates [30,31,32].

The aim of this study was first to analyze the temporal trend of NSIs rate among ward nurses and nurse assistants who worked for a period of ten years in a Northern Italian hospital during the progressive introduction into the healthcare setting of safety devices to replace conventional devices. Moreover, we explored the impact of rotating shift schedule, turnover, downsizing as well as staffing characteristics in terms of age and skill mix (job qualification level) on NSI rate trends.

## 2. Materials and Methods

This is an observational, dynamic cohort study of hospital workforce based on administrative hospital records. To determine injury types, we accessed the digital registry of occupational exposures to blood-borne infection injuries of a Northern Italian Hospital in Varese. The registry contains a standard set of information to define the type and severity of the injury, the results of blood testing for HBV, HCV and HIV serological markers of the source patient as well as of the injured personnel, at baseline and during follow-up if required. We selected injuries that occurred to nurses (N) and nurse assistants (NA) between 1 January 2007 and 31 December 2016, a period characterized by the progressive introduction of safety containers and procedures to avoid needle recap. Since 2008, passive safety devices have been adopted first in the wards with patients at higher risks of transferring blood-borne infections and subsequently in all hospital departments, following the Council Directive (2010/32/EU) on preventing sharp injuries in the healthcare environment. The devices adopted were: blunt-fill cannulae, vacuum-tube blood collection devices, safety winged butterfly steel needles, and safety containers. The introduction of the new devices was preceded in 2007, and subsequently repeated at two-year intervals, by a half-day training for all nurses and nursing assistants addressing the following topics: infectious risk for healthcare workers, on transmission of blood-borne pathogens; how to use the new passive safety devices and about the use of the specific procedure to timely report injury at occurrence.

To define the study population, we accessed the hospital payroll archives with demographic and occupational data including age, gender, job category, level of job qualification, hospital ward, shift schedule, and the number of effective worked hours (i.e., excluding non-working periods due to sickness absences, injuries, maternity leaves, and holiday periods). This information was provided for the entire investigated period, so we could track changes at an individual level. We were able to track new hirings/work cessations as well as internal transfers within hospital wards, used to calculate turnover and downsizing rates. We selected nurses and nurse assistants, and calculated the number of those exposed used as a denominator for the injury rates, as reported below. Hospital wards are grouped into three main areas: emergency department (ED-IC); medical and surgical wards. The shift schedules used for the analysis were: day shift only (permanent morning/day shift), day/evening (alternating between morning and afternoon/evening shifts), day/evening/night (rotating between morning, evening, and night shifts). We excluded nurses and nurse assistants working in surgery rooms and outpatient-dedicated clinical services, as they work on night shift occasionally and are not based on fixed shifts. The study was approved by the Hospital of Varese Ethics Committee (study ID 233/2019, 14 January 2020).

We synthesized age and prevalence of gender groups, working hours, hospital ward, and job category using standard descriptive statistics in the overall sample as well as by shift schedule. We tested the null hypotheses of no differences across shift schedule workers using either the Wilcoxon rank test or Chi-square test for continuous and discrete variables, respectively. We estimated the injury rates per 100 Full-Time Equivalent workers (FTEs) and relative 95% Confidence Intervals by shift schedule from Poisson regression models adjusted for age and job category in the overall sample and stratified by job category, hospital ward, and injury type. FTEs were computed from the effectively worked hours as defined above, considering 36 h/week as full time. We estimated the Annual Percent Change (APC) in injury rates using the joinpoint regression model (software: Joinpoint Regression Program, National Cancer Institute, Bethesda, Montgomery, MA, USA, V.4.0.4). The joinpoint approach allows estimating the number and the position of turning points in time trends, if any, using the Bayesian Information Criterion. To characterize the organizational factors, we computed the following indicators: turnover rate, as the total number of personnel changes every 100 FTEs in a given period; up/down sizing rate, as the difference in the working population at the beginning and the end of a period, every 100 FTEs. Staffing models indicators were: age mix, as the prevalence ratio between workers younger vs. older than the median age; skill mix, as the prevalence ratio between low vs. high level of achieved professional job qualification. Relevant data come from the hospital administrative databases. These indicators were computed for the hospital wards included in the study and separately for job category and shift schedule. We reported each indicator at three time points, i.e., at the beginning of the study period (2008); at the turning point selected by the joinpoint regression model (2012); and at the end of the study period (2016). Of note, 2008 represents the first year with available data in the hospital administrative databases. We tested the null hypothesis of no change over time in these indicators using either log-linear, linear and log-binomial regression models for turnover, up/down sizing, and age and skill mix, respectively. Statistical significance was set at the standard alpha level of 0.05. Statistical analyses other than joinpoint modelling were performed using SAS software version 9.4.

## 3. Results

The study population comprised 1615 and 1679 nurses and nurse assistants combined at the beginning and at the end of the study period, respectively. During the ten years of observation, 759 injuries were notified, 70.5% were percutaneous injuries, and 83.3% occurred to nurses. As shown in Table 1 (panel a) in the observed period, considering both Ns and NAs together, PI rates steeply decreased from 2007 to 2012, and increased in the subsequent years. This downward trend did not occur for mucocutaneous injury rates over the investigated period, with fluctuations due to the low number of events. Disaggregating PI rates for Ns and NAs (Table 1, panel b), only for the latter group, a monotonic decreasing trend was evident over the entire period, with rates starting from 3.9 per 100 FTE in 2007 and reaching 1.5 per 100 FTE in 2015 and 2016. Among nurses, PI rates were 7.6 per 100 FTE in 2007, reached a minimum value of 2.6 in 2012, and again rose until stabilized above 4.0 per 100 FTE in 2013–2016. As reported in Supplementary Materials Table S1, more than 95% of the PIs are NSIs, both in Ns and NAs.

The demographic and occupational characteristics of the study sample at the end of the observation period (2016), overall and by shift schedule, are shown in Table 2. The sample consisted of 1213 Ns and 466 NAs, who were mostly female (81.4%) and working full time (81.7%). Nearly three-quarters of the Ns and half of the NAs worked on a day/evening/night shift schedule. The median age was 45.3 years, and personnel on a day/evening/night shift schedule were in median about 8.3 years younger than those on a day shift only and day/evening shift. Also, Ns and NAs working on a day/evening/night shift schedule were more likely to be men, less likely to be on a part-time job, and more frequently working in ED-IC, than the other two shift-schedule-based groups. Of note, total FTEs based on effective worked hours were 1198.2, 71% of the total sample. This number represents the effective time of exposure to injuries, supporting the notion that injury rates based on the number of workers may be severely underestimated.

The overall PI rate, adjusted by age and job category, was 3.9 injuries per 100 FTE (95% CI: 3.6, 4.3), with no significant differences across shift schedules (*p*-value = 0.11; Table 3). Injury rate was higher in Ns (4.4; 3.9, 5.0) than in NAs (2.8; 2.3, 3.5), but there were no differences by shift schedule in either job categories. While no differences in injury rates by shift schedule were observed in ED-IC and medical wards, in surgical wards we found a lower injury rate among day-shift-only personnel (1.2; 0.6, 2.8) compared to the other two shift groups.

**Figure 1:**
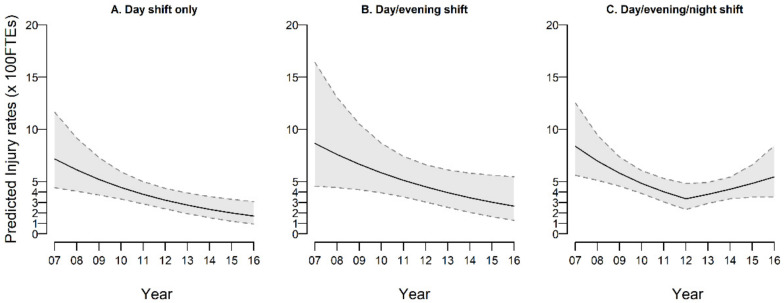
Predicted time trends (with 95% confidence bounds) for percutaneous injury rates in nurses, by shift schedule. In the plot: predicted time trend (line) with 95% confidence bounds (grey area).

The joinpoint regression analysis estimated one turning point in 2012 for the time trend in PI rates, with an APC 2007–2012 of −15.8% (95% CI: −23.6%, −8.0%) and a tendency to increase thereafter [APC in 2012–2016 = 2.4% (95% CI −8.4%, 13.1%)] (Table 4). Stratifying by job categories, the turning point was located in 2012 for nurses only, with an APC 2007–2012 of −18.5% (95% CI: −27.6%; −9.5%), and a subsequent increase tendency [APC 2007–2012 = 7.7% (−5.0%; 20.4%)]. A continuous decreasing trend of PI rates in NAs is confirmed over the entire period [−15.4% (−32.9%; 2.2%)]. Finally, when looking at trend of PIs in nurses only, and stratifying by shift schedule, a consistent and continuous decline over time was found for day shift only [APC: −20.9% (−29.8%, −12.0%)] and in part for day/evening shift (−12.8%; −30.9%, 5.4%) nurses. Conversely, in nurses on day/evening/night shift, the APC significantly reduced in 2007–2012 (−19.4%; −27.9%, −10.9%) followed by a significantly increased APC thereafter (APC: +13.5%; 1.5%, 25.5%). These trends are illustrated in Figure 1, while the underlying observed and estimated rates are reported in Supplementary Materials Table S2.

Table 5 reports the analysis of differences across shift schedules in indicators of organizational work characteristics calculated in the years 2008, 2012 (joinpoint detected by the model), and 2016. For turnover and up-down sizing, the table shows the absolute rate for 100 FTEs (see methods), while for the age mix and the skill mix, we report the prevalence ratio between the percentages of younger staff and less-experienced staff, respectively, having the day shift only as the reference category. Turnover rate shows a significant decline between 2008 and 2016 in day shift only Ns, a group that shows higher downsizing rates over the entire study period. The combination of both evidences support the interpretation that a reduction in number and a stabilization of such nurses occurred. In comparison to day shift only, a larger proportion of younger nurses was observed in the other two groups in the three time points. However, in Ns assigned to day/evening/night shift schedule, the prevalence ratio rose from 3.2 in 2008 to 5.9 in 2016 (*p*-value for trend: 0.04), meaning that younger Ns in this group are 6-fold more likely than in the day shift only. In the same year 2016, a higher percentage of less-experienced nurses (i.e., nurses with a lower level of professional qualification) was assigned to the day/evening/night shift schedule compared to the day-shift-only schedule (prevalence ratio = 2.5), a value much higher than in the two earlier years (*p*-value for trend < 0.0001).

## 4. Discussion

During the observation period, the overall PI rates declined steadily by 15.8% annually in the first six years (2007–2012), a decline that cannot be explained by chance only, nor by systematic and progressive underreporting. In that time period, passive safety procedures, based on the adoption of safety containers and procedures to avoid needle recap, were introduced and progressively adopted in the investigated hospital. This interpretation is supported by the evidence that mucocutaneous injury rates did not change. In more recent years (2013–2016), substantially no changes occurred in PI rates. Among safety devices that have been shown to lower NSI rates [9,10,11,12], safety containers that allow to avoid needle recap have shown better effectiveness in decreasing NSIs rates than devices with more sophisticated active safe mechanisms [13,14]. Our results support these previous findings.

A second major result of the present study is that when we stratified by Ns and NAs, the downward trend in PI rates was observed up to 2012 in Ns and over the entire observational period among NAs. The evidence of a turning point in PI rate trend in 2012 in Ns only required further investigations, focusing first on different shift schedules. We found that in day Ns the reduction in PI rates was monotonically present in the entire period. Conversely, in Ns engaged in the day/evening/night shift, we observed a significant reduction in rates of 19.4%/year between 2007 and 2012, followed by a statistically significant increase thereafter of 13.5%/year. Further investigating possible explanations, we evaluated time differences across shift schedule groups in organizational conditions during the same period, i.e., turnover and downsizing rate as well as staffing models indicators, i.e., age mix and skill mix. From these analyses, it appears that in the later years (2012–2016) a combination of organizational constraints occurred for nurses assigned to a day/evening/night shift schedule as compared to day shift only: they were more likely to be young and with less skill qualification. These characteristics, combined with the known effects of sleep deprivation on skill performance, suggest the need of organizational interventions to avoid taking blood samples by nurses at the end of the night shift. Our findings confirm previous studies carried out on American military nurses that have pointed out how a lower percentage of experienced staff might be associated with a higher NSI occurrence and that the right mix of skilled versus less-skilled nurses might be important in terms of staff-related outcomes [32,33]. In relation to shift schedule and NSIs in nurses, a few previous studies have found a correlation between rotating shifts and higher NSI rates [24,25], while others have found no associations between shift work and NSIs [23,34,35]. Our results show that it might not be shift work per se, but rather shift-level organizational factors and staffing models that influence NSIs trends and that they may even interfere with the effectiveness of passive safety devices in hospital ward nurses.

A few potential limitations need to be taken into account. First, the study design does not allow the interpretation of the association between change in organizational factors and change in injury rates in a causal way. To this extent, future studies with appropriate quasi-experimental designs are needed. Second, injury time was not consistently collected during the study period, to allow for analyzing injury rates by time of the day. Third, occupational exposures to blood-borne pathogens and injury trends can be affected by underreporting. In our study, injuries were drawn from a hospital dedicated register, based on a revised procedure established from 2004 on. As reported in Methods, in the investigated period, training courses were provided to nurse coordinators and nurse staff to assure knowledge of the correct use of the adopted safety devices and to continue to timely report injury occurrences. This contributes to reduce changes in underreporting of needlestick injuries over time. Moreover, the hospital policy allows the medical follow-up of injured personnel only in the case of registered injury. The medical follow-up consisted in a clinical examination by an infectious diseases specialist, who can request more in-depth investigations and provide antiretroviral post-exposure prophylaxis, if needed; as well as in monitoring of blood determinations to early detect blood-borne infections and injury insurance coverage. In addition, the consistency of our findings for different kinds of injuries and different job titles is an index of external consistency. We assume that there might be a run-in period with some underreporting when the new registration system was started in the study hospital in 2004–2006, and for this reason, those years were excluded from the current analysis. Finally, the availability of longitudinal data at an individual level on shift schedule, hospital ward and effective time spent at work could have allowed a more precise estimate of the denominator-at-risk of injury, avoiding potential underestimate of injury rates.

Several previous studies have analyzed shift schedules, organizational factors, staffing models and the adoption of safety devices in relation to NSIs in nurses. We further elaborate on these concepts and highlight their potentially intertwined and contrasting effects on NSIs trends in nurses by showing that the adoption of safety devices might not be equally successful across shift schedules when differences in shift-level staffing models are not taken into account. Our findings indicate the need for an integrated approach to NSIs prevention initiatives among hospital nurses.

Finally, based on our study, we may recommend careful consideration of multiple exposures and organizational procedures when assessing time trends of needlestick injury rates in the real field. Even if it is not easy, their control in designing and conducting both interrupted time series and comparative time series studies may probably help in interpretations of findings and increase the quality of evidence [13] of future investigations.

## 5. Conclusions

In a ten-year study period characterized by the progressive adoption of safety devices, we observed a steady decline in percutaneous injury rates among nurses and nurse assistants. In more recent years, among night-shift Ns only, we observed an opposite upward trend of IRs. This unexpected finding can be attributed to the combination of staffing factors (higher proportions of younger and less-experienced personnel involved in night-shift work in these years) with organizational constraints and practices such as taking blood-drawing at the end of the night shift. Moreover, our findings highlight the need to take carefully into consideration changes over time of organizational procedures and constraints when assessing time trends of needlestick injury rates in the real field, to avoid misinterpretations in the estimates of the effects of safety devices.

## Figures and Tables

**Table 1:** Number of injuries and injury rates (per 100 FTE) and 95% CI per year, among nurses and nurse assistants in the observed period, by: (a) injury type; and (b) job category, percutaneous injuries only.

Panel A	Percutaneous Injuries					Mucocutaneous Injuries				
	N events	FTE/year	Rate	95% CI		N events	FTE/year	Rate	95% CI	
2007	76	1166.3	6.5	5.1	8.0	22	1166.3	1.9	1.1	2.7
2008	74	1206.9	6.1	4.7	7.5	25	1206.9	2.1	1.3	2.9
2009	72	1229.1	5.9	4.5	7.2	29	1229.1	2.4	1.5	3.2
2010	60	1240.8	4.8	3.6	6.1	26	1240.8	2.1	1.3	2.9
2011	43	1255.8	3.4	2.4	4.4	19	1255.8	1.5	0.8	2.2
2012	34	1245.3	2.7	1.8	3.6	28	1245.3	2.2	1.4	3.1
2013	46	1190.5	3.9	2.7	5.0	14	1190.5	1.2	0.6	1.8
2014	48	1230.3	3.9	2.8	5.0	27	1230.3	2.2	1.4	3.0
2015	41	1240.1	3.3	2.3	4.3	20	1240.1	1.6	0.9	2.3
2016	41	1234.5	3.3	2.3	4.3	14	1234.5	1.1	0.5	1.7
**Panel B**	**Nurses**					**Nurse Assistants**				
	**N events**	**FTE/year**	**Rate**	**95% CI**		**N events**	**FTE/year**	**Rate**	**95% CI**	
2007	63	831.3	7.6	5.7	9.4	13	335.0	3.9	1.8	6.0
2008	65	855.3	7.6	5.8	9.4	9	351.5	2.6	0.9	4.2
2009	56	886.9	6.3	4.7	8.0	16	342.2	4.7	2.4	7.0
2010	48	896.1	5.4	3.8	6.9	12	344.8	3.5	1.5	5.4
2011	31	896.6	3.5	2.2	4.7	12	359.3	3.3	1.5	5.2
2012	23	895.7	2.6	1.5	3.6	11	349.6	3.1	1.3	5.0
2013	44	858.0	5.1	3.6	6.6	2	332.5	0.6	0.0	1.4
2014	42	892.3	4.7	3.3	6.1	6	338.0	1.8	0.4	3.2
2015	36	902.1	4.0	2.7	5.3	5	338.0	1.5	0.2	2.8
2016	36	898.8	4.0	2.7	5.3	5	335.7	1.5	0.2	2.8

FTE: Full-Time Equivalent, computed considering 36 h/week and from the total amount of working hours during the year 2016.

**Table 2:** Characteristics of the study population at the end of the observation period (2016), by shift schedule.

	All Sample	Shift Schedule			*p*-Value
		Day Shift Only	Day/Evening Shift	Day/Evening/Night Shift	
**No. of individuals**	1679	196	383	1100	-
**FTE/years**	1198.2	130.3	255.4	812.4	-
**Age ^**	45.3 (35.7; 52.8)	49.9 (46.2; 55.2)	51.0 (43.9; 56.2)	41.3 (32.4; 49.7)	<0.0001
**Women, n (%)**	1366 (81.4)	182 (92.9)	345 (90.1)	839 (76.3)	<0.0001
**Working hours, n (%)**					
* Full time*	1372 (81.7)	128 (65.3)	278 (72.6)	966 (87.8)	<0.0001
* Part time*	307 (18.3)	68 (34.7)	105 (27.4)	134 (12.2)	
**Hospital wards, n (%)**					
* ED-IC*	427 (25.4)	27 (13.8)	66 (17.2)	334 (30.4)	<0.0001
* Medical ward*	772 (46.0)	114 (58.2)	188 (49.1)	470 (42.7)	
* Surgical ward*	480 (28.6)	55 (28.1)	129 (33.7)	296 (26.9)	
**Job category, n (%)**					
* Nurses*	1213 (72.3)	151 (77.0)	190 (49.6)	872 (79.3)	<0.0001
* Nurse assistants*	466 (27.7)	45 (23.0)	193 (50.4)	228 (20.7)	

FTE: Full-Time Equivalent, computed considering 36 h/week and from the total amount of working hours during the year 2016. ^: Median (25–75° Percentiles). Abbreviations: ED-IC = Emergency Department and Intensive Care.

**Table 3:** Number of percutaneous injuries and injuries rates (per 100 FTEs) with 95% confidence intervals, according to shift schedule, in the overall sample and stratified by job category and hospital ward in the entire observed period (2007–2016).

	No. of Injuries	FTE	Injury Rates (95% CI) ^	Injury Rates by Shift Schedule (95% CI) ^			*p*-Value §
				Day Shift Only	Day/Evening Shift	Day/Evening/Night Shift	
**All sample**	535	12239.8	3.9 (3.6; 4.3)	3.2 (2.4; 4.3)	4.6 (3.8; 5.5)	3.9 (3.4; 4.4)	0.11
**Job category, n (%)**							
* Nurses*	444	8813.2	4.4 (3.9; 5.0)	3.9 (2.9; 5.3)	4.9 (3.8; 6.2)	4.4 (3.9; 5.1)	0.55
* Nurse assistants*	91	3426.6	2.8 (2.3; 3.5)	1.5 (0.6; 3.6)	3.5 (2.6; 4.7)	2.5 (1.8; 3.5)	0.07
**Hospital wards, n (%)**							
* ED-IC*	143	3327.4	3.7 (3.1; 4.5)	2.2 (0.9; 5.4)	4.4 (2.9; 6.8)	3.8 (3.0; 4.7)	0.35
* Medical ward*	244	5201.7	4.5 (3.9; 5.1)	4.7 (3.4; 6.4)	5.3 (4.2; 6.8)	3.8 (3.2; 4.6)	0.09
* Surgical ward*	148	3710.7	3.5 (2.9; 4.2)	1.2 (0.6; 2.8)	3.4 (2.3; 5.2)	3.9 (3.1; 5.0)	0.005

FTE: Full-Time Equivalent, computed considering 36 h/week and from the total amount of working hours during the year 2016. ^: Rates are adjusted by age and job category, estimated with Poisson regression models. §: Likelihood ratio Chi-square test for heterogeneity of injury rates across shift schedule categories (2 df), from Poisson regression models. Abbreviations: ED-IC = Emergency Department and Intensive Care.

**Table 4:** Joinpoint analysis of trends in percutaneous injury rates in 2007–2016. Annual Percent Change (APC) and 95% CI are reported for the entire period or for two periods in case of joinpoint(s) ^.

Periods	2007–2016	
	2007–2012	2012–2016
**Overall**	−15.8 (−23.6; −8.0)	2.4 (−8.4; 13.1)
**by job title**		
*Nurses*	−18.5 (−27.6; −9.5)	7.7 (−5.0; 20.4)
*Nurse assistants*	−15.4 (−32.9; 2.2)	
**in nurses, by shift schedule**		
*Day shift only*	−20.9 (−29.8; −12.0)	
*Day/evening shift*	−12.8 (−30.9; 5.4)	
*Day/evening/night shift*	−19.4 (−27.9; −10.9)	13.5 (1.5; 25.5)

^: APCs and 95% confidence intervals estimated by the joinpoint regression model. The number and the position of joinpoints selected by the model using the Bayesian Information Criteria method.

**Table 5:** Turnover and up-down sizing rates (per 100 FTEs), and prevalence ratios ^ for age and skill mixes, among study nurses, by day shift scheme. Years 2008, 2012 and 2016, and *p*-values to assess time trends.

	Period, Year			*p*-Value Trend Test	
	2008	2012	2016	2012 vs. 2008	2016 vs. 2008
**Turnover in hospital wards °**					
* Day shift only*	50.6	40.8	**24.4**	0.2	**0.0003**
* Day/evening shift*	40.8	35.4	31.4	0.4	0.2
* Day/evening/night shift*	38.6	40.2	44.2	0.6	0.1
**Up-down sizing in hospital wards °**					
* Day shift only*	−5.2	−14.4	−4.9	0.4	1.0
* Day/evening shift*	0.5	1.7	3.8	0.9	0.8
* Day/evening/night shift*	0.2	3.1	−0.7	0.6	0.9
Age mix *					
* Day shift only*	ref	ref	ref	-	-
* Day/evening shift*	2.5 (1.8; 3.6)	2.2 (1.5; 3.3)	3.2 (1.9; 5.3)	0.7	0.5
* Day/evening/night shift*	3.2 (2.7; 4.4)	3.4 (2.4; 4.7)	**5.9 (3.6; 9.5)**	0.8	**0.04**
Skill mix **					
* Day shift only*	ref	ref	ref	-	-
* Day/evening shift*	1.4 (1.2; 1.6)	1.4 (1.2; 1.5)	1.9 (1.5; 2.4)	0.6	0.06
* Day/evening/night shift*	1.5 (1.3; 1.7)	1.4 (1.3; 1.6)	2.5 (2.0; 3.2)	0.6	<0.0001

**°**: Turnover and up-down sizing expressed as rate per 100 FTEs. * Age mix: younger vs. older age prevalence ratio, where younger age is defined as age below the median age, specific for each year. ** Skill mix: low vs. high experience level prevalence ratio, where low experience is measured from professional qualification level. ^: Prevalence ratios and 95% confidence intervals (in brackets), with day shift as reference category, estimated from log-binomial models.

## Data Availability

Individual-level data used in this study are not publicly available, as they are owned by the ASST-Sette Laghi Varese hospital administration. Aggregated data may be available upon request to the corresponding author.

## Supplementary Materials

The following are available online at https://www.mdpi.com/article/10.3390/ijerph18084371/s1, Table S1: Number of percutaneous injuries, by injury agent and year, in the overall sample of Health Care Workers (left) and further divided by job title (nurses and nurse assistants). Table S2: Number of injuries, full-time equivalent (FTE), observed injury rates per 100 FTE (with 95% CI) and predicted injury rates for percutaneous injuries, per year, in nurses, by shift schedule.
