# Supplementary material for: Time Trends of Percutaneous Injuries in Hospital Nurses: Evidence of the Interference between Effects of Adoption of Safety Devices and Organizational Factors
Source: Int J Environ Res Public Health. 2021 Apr 20;18(8):4371. doi: 10.3390/ijerph18084371 (PMC8074301; doi:10.3390/ijerph18084371)
Supplement: Supplementary file 1 [file ijerph-18-04371-s001.zip › ijerph-1157916-supplementary.pdf]

Time trends of percutaneous injuries in hospital nurses: evidence of the interference between effects of adoption of safety devices and organizational factors.

Marco M. Ferrario[1,2,], MD; Giovanni Veronesi[1], PhD; Rossana Borchini[3], MD; Marco Cavicchiolo[4], MD; Oriana Dashi[4], MD; Daniela Dalla Gasperina[1], MD; Giovanna Martinelli[5;] and Francesco Gianfagna[1,6], MD.

[1] Department of Medicine and Surgery, School of Medicine, University of Insubria, Varese, Italy
[2] Occupational, Preventive and Toxicology Unit, ASST Sette Laghi, Varese, Italy
[3] Occupational and Preventive Medicine Unit, ASST Lariana, Como, Italy
[4] School of Specialization in Occupational Medicine, University of Insubria, Varese, Italy
[5] Quality Health Assessment Unit, ASST Sette Laghi, Varese, Italy
[6] Mediterranea Cardiocentro, Napoli, Italy

Supplementary Tables

Table S1: Number of percutaneous injuries, by injury agent and year, in the overall sample of Health Care Workers (left) and further divided by job title (nurses and nurse assistants).

Year	All HCW					Nurses					Nurse assistants				
	Percutaneous injuries	Needles		Scalpels/other sharp device		Percutaneous injuries	Needles		Scalpels/other sharp device		Percutaneous injuries	Needles		Scalpels/other sharp device	
		n	%^	n	%^		n	%^	n	%^		n	%^	n	%^
2007	76	74	97.4	2	2.6	63	62	98.4	1	1.6	13	12	92.3	1	7.7
2008	74	72	97.3	2	2.7	65	64	98.5	1	1.5	9	8	88.9	1	11.1
2009	72	69	95.8	3	4.2	56	53	94.6	3	5.4	16	16	100.0	0	0.0
2010	60	56	93.3	4	6.7	48	44	91.7	4	8.3	12	12	100.0	0	0.0
2011	43	37	86.0	6	14.0	31	29	93.5	2	6.5	12	8	66.7	4	33.3
2012	34	33	97.1	1	2.9	23	22	95.7	1	4.3	11	11	100.0	0	0.0
2013	46	42	91.3	4	8.7	44	40	90.9	4	9.1	2	2	100.0	0	0.0
2014	48	46	95.8	2	4.2	42	41	97.6	1	2.4	6	5	83.3	1	16.7
2015	41	37	90.2	4	9.8	36	32	88.9	4	11.1	5	5	100.0	0	0.0
2016	41	36	87.8	5	12.2	36	31	86.1	5	13.9	5	5	100.0	0	0.0

^: % on the total number of percutaneous injuries, by year.

Table S2: Number of injuries, full time equivalent (FTE), observed injury rates per 100 FTE (with 95%CI) and predicted injury rates^ for percutaneous injuries, per year, in nurses, by shift schedule

Year	Day shift only						Day/evening shift						Day/evening/night shift					
	Inj. No.	FTE	Observed rate	Observed 95%CI		Predicted rate	Inj. No.	FTE	Observed rate	Observed 95%CI		Predicted rate	Inj. No.	FTE	Observed rate	Observed 95%CI		Predicted rate
2007	8	112.3	7.1	2.2	12.1	7.2	11	131.6	8.4	3.4	13.3	8.7	44	587.4	7.5	5.3	9.7	8.4
2008	4	108.7	3.7	0.1	7.3	6.1	10	129.4	7.7	2.9	12.5	7.6	51	617.2	8.3	6.0	10.5	7.0
2009	9	118.4	7.6	2.6	12.6	5.2	11	129.1	8.5	3.5	13.6	6.7	36	639.5	5.6	3.8	7.5	5.8
2010	4	119.5	3.3	0.1	6.6	4.4	12	140.5	8.5	3.7	13.4	5.8	32	636.0	5.0	3.3	6.8	4.8
2011	6	123.3	4.9	1.0	8.8	3.8	2	126.5	1.6	0.0	3.8	5.1	23	646.8	3.6	2.1	5.0	4.0
2012	5	118.9	4.2	0.5	7.9	3.2	3	128.8	2.3	0.0	5.0	4.5	15	648.1	2.3	1.1	3.5	3.4
2013	2	112.1	1.8	0.0	4.3	2.7	7	123.3	5.7	1.5	9.9	3.9	35	622.6	5.6	3.8	7.5	3.8
2014	4	116.1	3.4	0.1	6.8	2.3	5	126.1	4.0	0.5	7.4	3.5	33	650.1	5.1	3.3	6.8	4.3
2015	2	116.9	1.7	0.0	4.1	2.0	2	127.9	1.6	0.0	3.7	3.0	32	657.3	4.9	3.2	6.6	4.8
2016	1	104.4	1.0	0.0	2.8	1.7	5	126.6	4.0	0.5	7.4	2.7	30	667.8	4.5	2.9	6.1	5.5

FTE: Full-Time Equivalent, computed considering 36 hrs/week and from the total amount of working hours during the year 2016.
^: predicted injury rates: from joinpoint regression model, with a turning point at 2012 for nurses in day/evening/night shift.
